# Epidemiology and Antithrombotic Therapy for Cancer-Associated Arterial Thromboembolism in Japan∗

**DOI:** 10.1016/j.jaccao.2024.02.002

**Published:** 2024-04-16

**Authors:** Wei Xiong, Yugo Yamashita

**Affiliations:** a Department of Cardiovascular Medicine, Graduate School of Medicine, Kyoto University, Kyoto, Japan; b Department of Pulmonary and Critical Care Medicine, Xinhua Hospital, Shanghai Jiaotong University School of Medicine, Shanghai, China

**Keywords:** anticoagulant, artery, cancer, thrombosis

**Figure:**
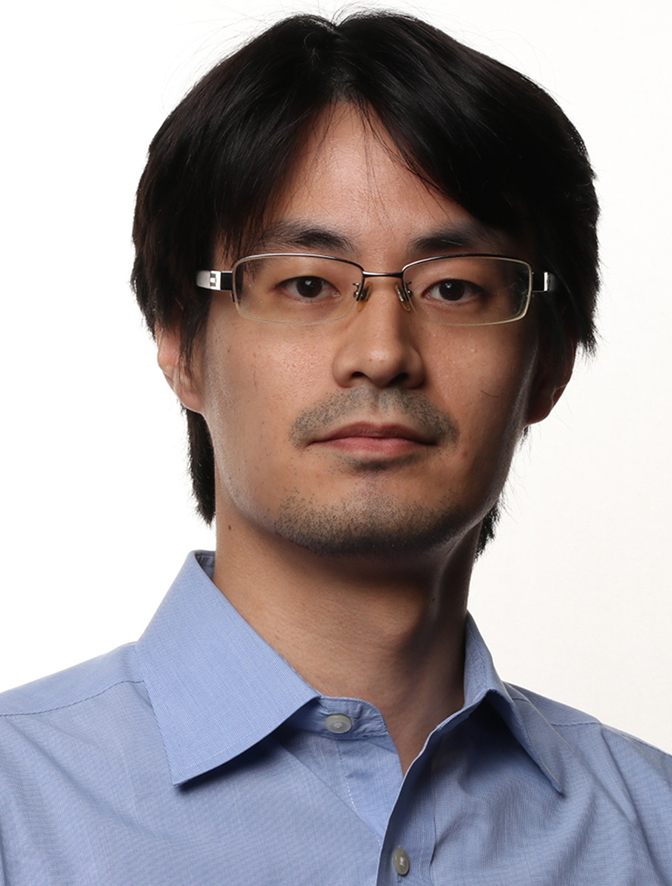

Cancer-associated thrombosis (CAT) is the second leading cause of mortality following cancer itself in patients with cancer.^1^ Although venous thromboembolism is the most common type of CAT, arterial thromboembolism (ATE), including myocardial infarction and ischemic stroke, is also prevalent.^2^ Although there are several clinical guidelines for cancer-associated venous thromboembolism,^3^, ^4^, ^5^ there have been no established clinical guidelines for cancer-associated ATE to date. Because patients with cancer have a higher risk of bleeding events and a shorter life expectancy,^6^ antithrombotic therapy for cancer-associated ATE has been a matter of active debate. There is still uncertainty regarding the optimal management strategies of cancer-associated ATE.

Several previous studies reported the current epidemiologic data of cancer-associated ATE in Europe and the United States.^7^, ^8^, ^9^ However, there have been limited data on cancer-associated ATE in Asian countries. To address this need, in this issue of *JACC: CardioOncology*, Gon et al^10^ conducted a retrospective multicenter observational study using data from the Osaka Cancer Registry linked with administrative data between 2010 and 2015. They investigated the incidence of cancer-associated ATE, the predisposing risk factors of ATE development, and whether antithrombotic therapy improved prognosis in 97,448 Japanese patients with cancer. Among 97,448 cancer patients, a total of 2,159 patients developed cancer-associated ATE, and the incidence rate of cancer-associated ATE was 1.29% at 1 year after cancer diagnosis. The incidence rate during the first year after cancer diagnosis accounted for more than half of the incidence rate during the 5 years after cancer diagnosis, which is consistent with previous studies.^7^, ^8^, ^9^^,^^11^^,^^12^ The risk of cancer-associated ATE development varied considerably based on sex, age, cancer progression, and cancer type, which was also consistent with previous studies.^6^ Patients with cancer-associated ATE experienced a 2-fold increased risk of mortality compared with those without, suggesting the importance of cancer-associated ATE in terms of mortality risk. Overall, the clinical characteristics of cancer-associated ATE in Japan were similar to that of patients in Western countries.

The current study also provides valuable insight about the usefulness of antithrombotic therapy in cancer-associated ATE. Importantly, because the current study was an observational study, the effect of antithrombotic therapy on clinical outcomes in the current study should be interpreted cautiously as hypothesis generating. The authors showed that the 90-day and 1-year restricted mean survival time differences were 13.3 days (95% CI, 10.4-16.2; *P <* 0.001) and 57.8 days (95% CI: 43.1-72.5; *P <* 0.001) days with antithrombotic therapy, suggesting a favorable effect of antithrombotic therapy for cancer-associated ATE. Theoretically, cancer patients with ATE who receive antithrombotic therapy had a lower risk of thrombotic events than those who did not. Although the current study did not evaluate the impact of bleeding events related to antithrombotic therapy on mortality, the overall lower risk of mortality in these patients suggests that there may not be major concerns in patients who received antithrombotic therapy. Nevertheless, because the current study was an observational study with potential for limitations such as selection bias, randomized clinical trials would be needed to clarify the optimal management strategies of antithrombotic therapy for cancer-associated ATE patients.

In summary, Gon et al^10^ reported clinically valuable epidemiologic data on cancer-associated ATE, including the incidence, predisposing risk factors, and overall prognostic impact of ATE. In addition, the current study also provided important insights into the use of antithrombotic therapy in these patients. Interestingly, the characteristics of cancer-associated ATE in Japan seem to be largely similar to that from Western countries.

## Funding Support and Author Disclosures

Dr Yamashita has received lecture fees from Bayer Healthcare, Bristol-Myers Squibb, Pfizer, and Daiichi Sankyo; and has received grant support from Bayer Healthcare and Daiichi Sankyo. Dr Xiong has reported that he has no relationships relevant to the contents of this paper to disclose.
